# Stability of Mixed Lead Halide Perovskite Films Encapsulated in Cyclic Olefin Copolymer at Room and Cryogenic Temperatures

**DOI:** 10.1021/acs.jpclett.3c02733

**Published:** 2023-12-08

**Authors:** Mutibah Alanazi, Ashley Marshall, Shaoni Kar, Yincheng Liu, Jinwoo Kim, Henry J. Snaith, Robert A. Taylor, Tristan Farrow

**Affiliations:** † Clarendon Laboratory, Department of Physics, University of Oxford, Parks Road, Oxford, OX1 3PU, U.K.; ‡ Helio Display Materials Ltd., Wood Centre for Innovation, Oxford, OX3 8SB, U.K.; § Institute of Materials Research and Engineering, Agency for Science, Technology and Research (A*STAR), 2 Fusionopolis Way, Singapore 138634, Singapore

## Abstract

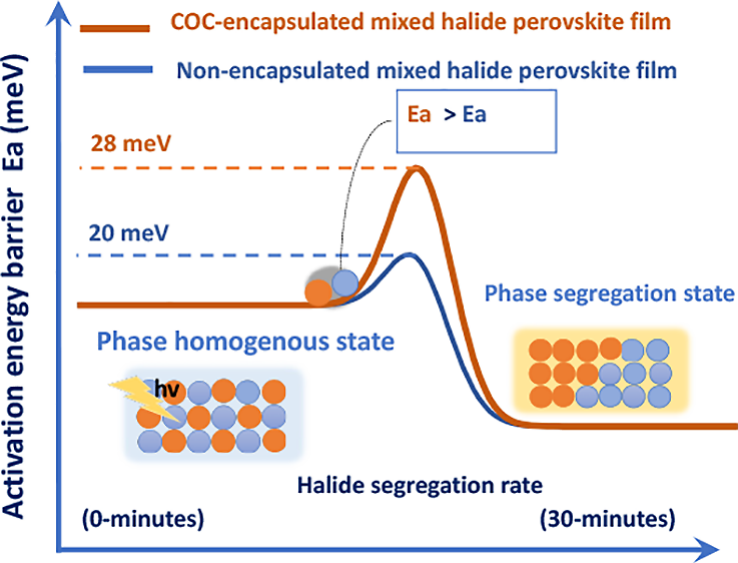

Lead Mixed Halide Perovskites (LMHPs), CsPbBrI_2_, have attracted significant interest as promising candidates for wide bandgap absorber layers in tandem solar cells due to their relative stability and red-light emission with a bandgap ∼1.7 eV. However, these materials segregate into Br-rich and I-rich domains upon continuous illumination, affecting their optical properties and compromising the operational stability of devices. Herein, we track the microscopic processes occurring during halide segregation by using combined spectroscopic measurements at room and cryogenic temperatures. We also evaluate a passivation strategy to mitigate the halide migration of Br/I ions in the films by overcoating with cyclic olefin copolymer (COC). Our results explain the correlation between grain size, intensity dependencies, phase segregation, activation energy barrier, and their influence on photoinduced carrier lifetimes. Importantly, COC treatment increases the lifetime charge carriers in mixed halide thin films, improving efficient charge transport in perovskite solar cell applications.

Recently, all-inorganic perovskites CsPbX_3_ (X = I, Br, Cl) have attained extensive attention due to their optical properties and thermal stability, making them promising candidates for practical applications when compared to their organic–inorganic perovskite counterparts.^1−4^ However, the CsPbI_3_ perovskite can readily convert from the cubic phase (α, β, γ-black phase) to the orthorhombic phase (δ-yellow phase) under ambient conditions, reducing photovoltaic performance given its large bandgap, high energy loss, and high defect density. Although CsPbBr_3_ shows excellent thermal stability,^5^ it has a bandgap of 2.3 eV, restricting its further development.^1,6^ Mixed halide perovskites CsPbI_3–*x*_Br_*x*_ enable the bandgap to be tuned across the entire visible spectrum.^7,8^ Furthermore, halide mixing can address phase transitions and produce stable perovskites at working temperatures, and the bandgap makes them appropriate for high-performance tandem solar cell top cells.^7^ CsPbBrI_2_ in particular has reasonable bandgaps in the range of (1.82–1.92 eV) with a wide light absorption range exhibiting great potential in tandem and semitransparent photovoltaic applications.^1,2,6,9^ It was shown that CsPbBrI_2_ is stable in the cubic phase at room temperature even for bulk materials.^10^ However, upon continuous illumination, iodide and bromide ions migrate in the CsPbBrI_2_ crystal lattice after obtaining sufficient energy and eventually accumulate at grain boundaries^9,11^ such that the ion accumulation leads to the formation of an I-rich phase (1.55 eV) and a Br-rich phase (2.3 eV), resulting in phase segregation deviating from the desired bandgap with red and blueshifts, respectively.^12^ This can negatively impact devices. Along with ion migration, current density–voltage (I–V) hysteresis and trap states can result in significant charge recombination, lower open circuit voltage, and lower fill factor (FF) values in devices based on mixed halide perovskite materials.

Several theoretical models have attributed the driving force for phase segregation/separation to (i) internal lattice strain,^13^ (ii) polaron-induced lattice strain,^14,15^ (iii) photocarrier energies,^16−19^ and (iv) the kinetics of halide vacancies. These are all discussed comprehensively in various reviews.^5,18^ The driving forces based on strain models are associated with an increase in shear strain in the perovskite lattice originating from either an internal lattice mismatch between halide ions or an external polaron-induced structural deformation.^3,14,20,21^ Consequently, halide ion segregation is more likely to occur to release the lattice strains, leading to photoinstability. Also, the relaxation and deformation of the structural lattice of the perovskite relieve the free energy, further promoting halide ion segregation.^4,21^ However, the strain-based consideration fails to consider the role of defects and photoexcited charge carriers in phase segregation.^14^ The photocarrier energy model involves the photogenerated charge carriers’ role in the rearrangement of halide ions within the perovskite lattice and their kinetic processes, including carriers’ generation, diffusion, and accumulation and recombination at I-rich domains.^17,18,22^ A bandgap difference between the initial and segregated phases can also drive halide segregation, increasing the free energy of the mixed halide in excited states.^19,20,23,24^ As in strain models, such an increase in the free energy needs to be minimized through halide segregation. The formation of halide vacancies inside the perovskite lattice can also be responsible for driving halide ion migration through defect-mediated movement. The migration is more likely to occur through halide Schottky defects or vacancies in the mixed halide perovskite lattice, which ultimately forms two stable Br-rich and I-rich phases under continuous illumination.^16,18,20,22^ More importantly, the lower activation energy for halide ion migration (0.17–0.43 eV for Br and I) than for cations (0.46–1.20 eV for Cs and 0.80–2.31 eV for Pb), and the resulting low formation energy of halide defects are mainly responsible for ion migration and generating a significant contribution to the ionic properties of MHPs.^13,16,23^ Thus, it is essential to take into consideration all of these processes simultaneously, including halide defect generation, halide ion migration, polaron formation, and any resulting phase segregation, as illustrated in Figure 1a–1b.^2−4^

**Figure 1:**
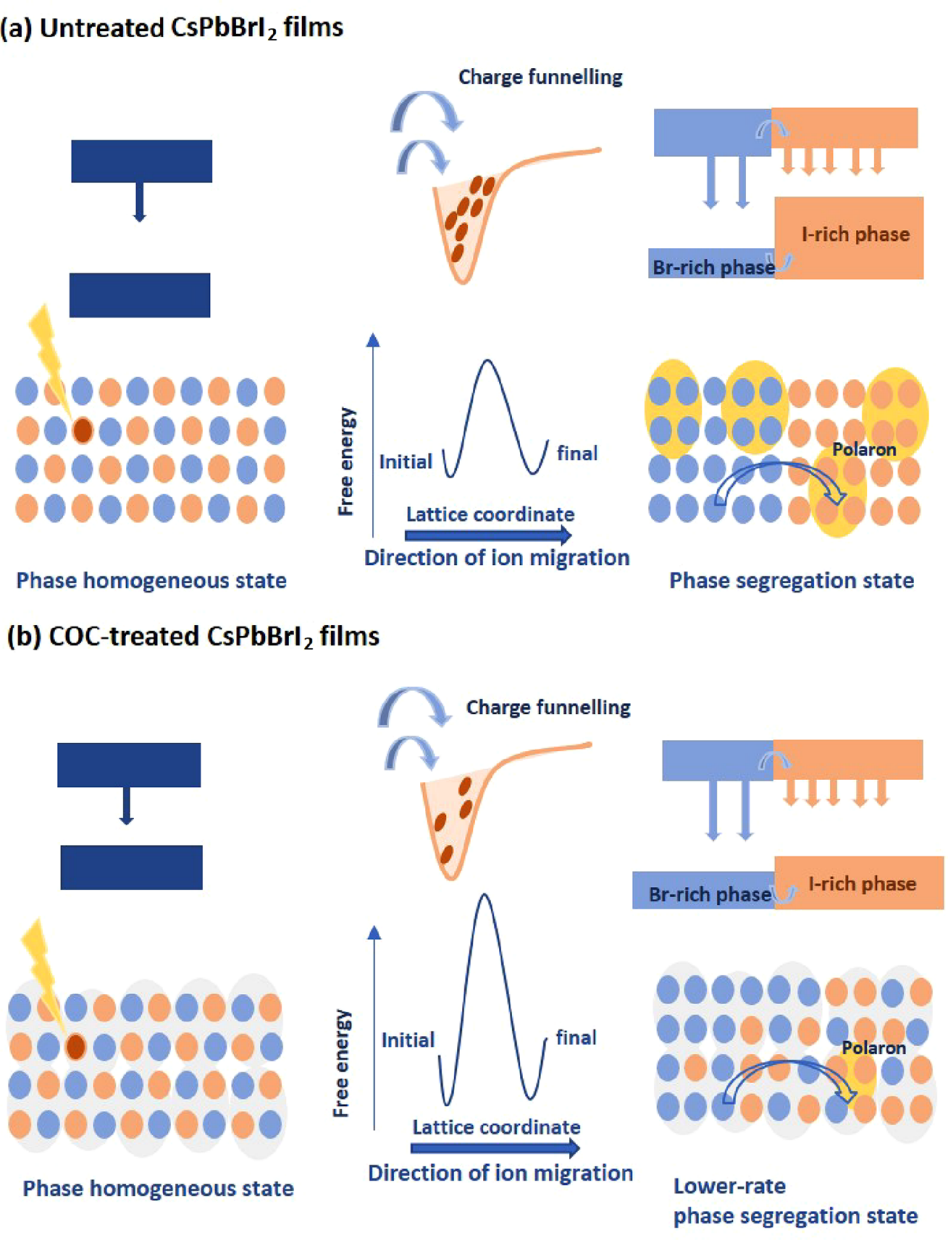
Schematics contrasting carrier and ion-migration dynamics in (a) untreated CsPbBrI_2_ films and (b) COC-treated CsPbBrI_2_ films.

In order to investigate these effects further, time-dependent PL at cryogenic temperatures was undertaken, where we expect less thermal lattice distortion and thereby lower polaron formation, as seen in Figures 3a–3d. In comparison to both films at room temperature in Figure 2a–2d, under prolonged illumination, a native broad peak stemming from bromide and iodide domains was observed at 685 nm in the untreated CsPbBrI_2_ films at 20 and 80 mW in Figures 3a–3b. Similarly, the predominant PL emission appeared at 675 nm in the COC-treated CsPbBrI_2_ films at 0.1 μW and 0.6 μW in Figures 3c–3d, indicating the absence of a difference in the bandgap and a photocarrier funneling effect. Thereby, entropy remixing of halide ions dominates the enthalpy (lattice strain) due to entropic stabilization. The COC-treated CsPbBrI_2_ films exhibited a narrower PL peak and blueshift of 5 nm compared to those of untreated CsPbBrI_2_ films for both excitation intensities. This suggests that COC leads to increased halide mixing and electronic transition homogeneity while the electron–phonon coupling strength becomes weaker compared to untreated films.^42^

**Figure 2:**
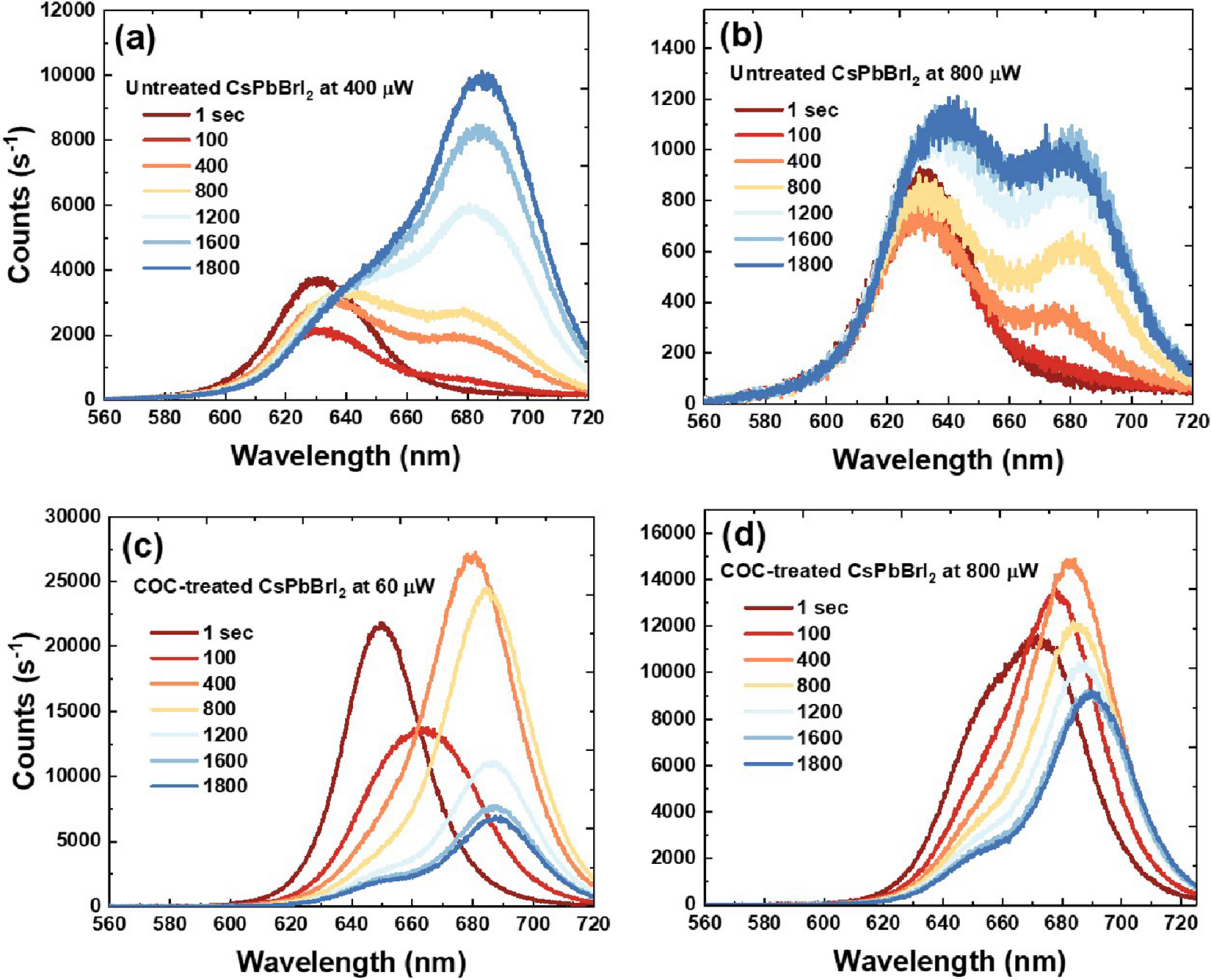
Room-temperature PL time-series for CsPbBrI_2_ films without COC and with COC treatment at low and high excitation powers. (a) Untreated CsPbBrI_2_ films at 400 μW, (b) untreated CsPbBrI_2_ films at 800 μW, (c) COC-treated CsPbBrI_2_ films at 60 μW, and (d) COC-treated CsPbBrI_2_ films at 800 μW.

**Figure 3:**
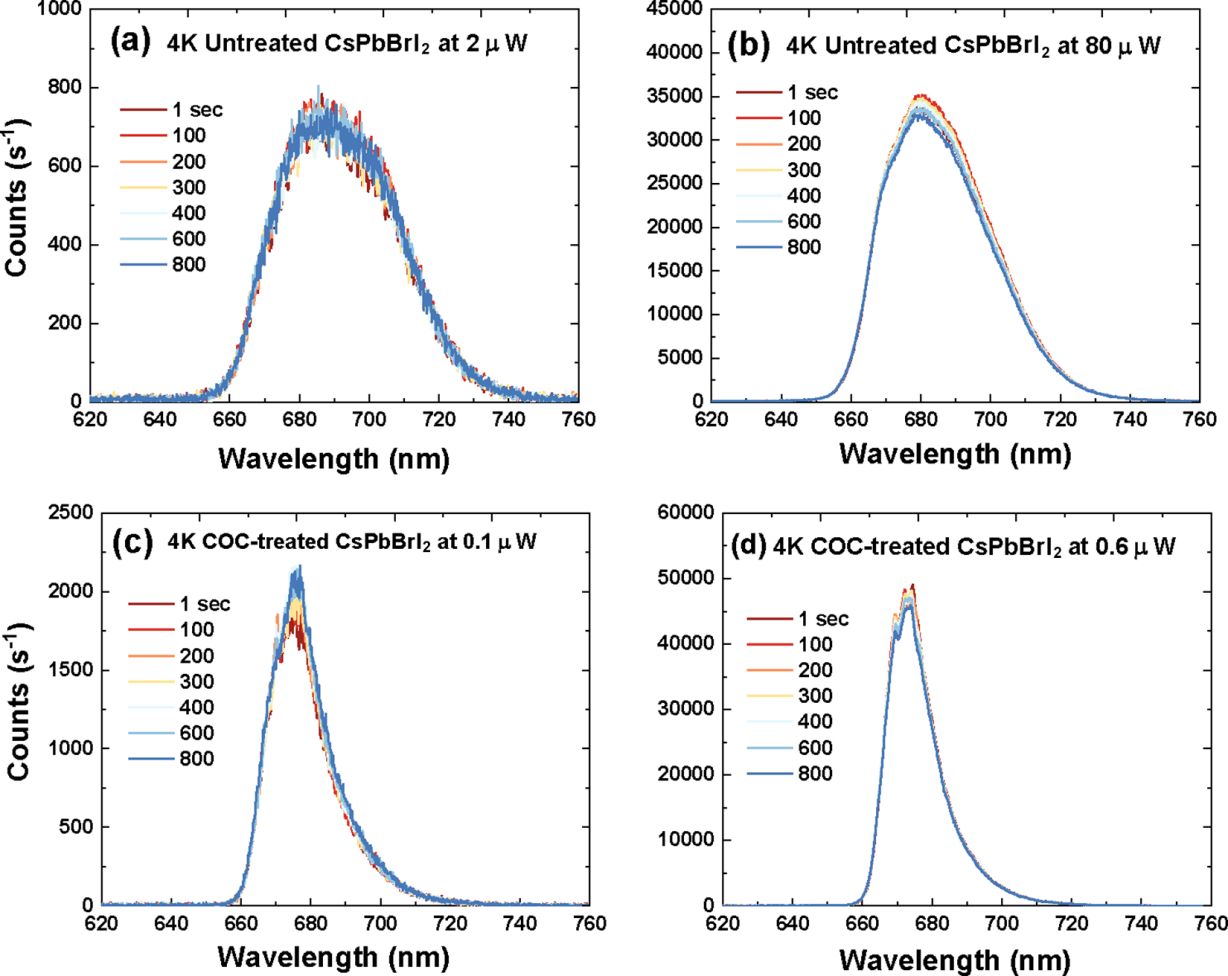
Cryogenic (4.2 K) PL time-series for CsPbBrI_2_ films without COC and with COC treatment at low and high excitation powers of (a) untreated CsPbBrI_2_ films at 52 μW, (b) pure CsPbBrI_2_ films at 80 μW, (c) COC-treated CsPbBrI_2_ films at 0.1 μW, and (d) COC-treated CsPbBrI_2_ films at 0.6 μW.

With regards to the PL peak center, the untreated CsPbBrI_2_ films showed a redshift of 55 nm from 630 nm at RT to 685 nm at 4.2 K, while COC-treated CsPbBrI_2_ films exhibit a smaller redshift of 30 nm from 645 nm at RT to 675 nm at 4.2 K. Unlike traditional II–VI chalcogenides, the all-inorganic perovskite materials exhibit a blueshift in PL emission with increasing temperature to 290 K.^42,43^ This might refer to the fact that the interplay between the electron–phonon renormalization and the thermal expansion has a reverse impact on the band gap energy. It is not expected to have any impact on phase segregation.^43,44^ Interestingly, the intensity of PL emission was increased without showing halide demixing with increasing excitation intensity for untreated and the COC-treated films in Figures 3b–3d, confirming the entropic preference for halide mixing.

The phase segregation and charge traps in perovskite materials have led to the development of efficient trap state passivation methods that enhance the photostability of perovskites. For instance, Yuan et al. report the addition of a Pb(NO_3_)_2_ methyl acetate solution to CsPbBrI_2_ perovskite film reduced the deep trap density from 8 × 10^16^ cm^–3^ to 6.64 × 10^16^ cm^–3^.^25^ Other groups proposed potassium bromide, PMMA, tri-iodine molecules, and trioctylphosphine oxide as passivation agents.^26−29^ After applying PMMA to CsPbI_3–*x*_Br_*x*_ micro platelets, Wang et al. found phase segregation was suppressed due to vacancy passivation at the perovskite surface.^29^ Yang et al. found that KBr passivation inhibited the PL shift in all-inorganic CsPbI_3–*x*_Br_*x*_ NCs, while the pristine counterpart showed phase segregation with PL peak shifting from 638 to 661 nm.^27^ Although the passivation of trap states in CsPbBrI_2_ film surfaces is considered a potential strategy for controlling halide segregation, the role of intrinsic defects in CsPbBrI_2_ perovskite and their passivation are still not fully understood.

In this work, we examine the validity of such strategies and the extent to which they can mitigate instability through the passivation of trap states using cyclic olefin copolymer (COC) treatment. Based on thermodynamic and kinetic models, we clarify the contributions that induce phase segregation in thin films of CsPbBrI_2_ mixed halide perovskites at room and cryogenic temperature. Our study probes experimentally the role of the energy barrier on halide migration, limiting lattice strain and polaron formation, and mitigating trap states. We implement a set of measurements involving room-temperature X-ray diffraction (XRD) characterization, temperature-dependent microphotoluminescence (PL) spectroscopy, room- and cryogenic-temperature (4.2 K) time-dependent PL profiles for increasing excitation intensities. This is further supported by time-resolved PL spectroscopy for coated and uncoated films to investigate the effects of the passivization method on the charge carrier dynamics.

Lead mixed halide perovskite CsPbBrI_2_ films were deposited on glass substrates by spin-coating perovskite precursors. The synthesis details and encapsulation method are described in the experimental section in the Supporting Information. The X-ray diffraction (XRD) characterization was first performed on the untreated CsPbBrI_2_ and the COC-treated CsPbBrI_2_ films to examine the crystal structure and calculate the grain size, as shown in Figures S1a–S1b. The position of diffraction peaks of 2θ at 15° and 30° remained unchanged, signifying the crystal structure of untreated CsPbBrI_2_ remains in the cubic phase after being treated with COC. However, the gradual narrowing of the diffraction peaks was observed in the untreated CsPbBrI_2_ films, indicating the enlarged grain size compared with the COC-treated CsPbBrI_2_ films. Such gradual narrowing has been attributed to the threshold size of phase segregation, which was equal to or larger than 43 nm. Here we implemented the Rietveld refinement XRD method to calculate the accurate average grain size. This allows us to compare our experimental findings to a calculated diffraction pattern based on an initial structural model. We then fitted the peak positions and narrowing/broadening to generate the Williamson–Hall plots to correctly estimate the average grain size and the effect of microstrain of untreated CsPbBrI_2_ films and the COC-treated CsPbBrI_2_ films. The average grain size calculated empirically by the Debye–Scherrer equation was 168 nm for untreated CsPbBrI_2_ films, and 47.33 nm for the COC-treated CsPbBrI_2_ as shown in Figures S2a–S2b. This trend is consistent with tabulated values in a previous study.^30^ This is supporting evidence that, above a threshold size of segregation which has been reported to be 43–46 nm in the literature,^30,31^ the diffusion length will be determined by the carrier’s natural diffusion lengths rather than by the grain size. We revisit this with time-resolved studies to confirm this result empirically.

To confirm the correlation between the grain size and phase segregation, a time-dependent PL profile was implemented on both films. Figures 2a–2d represent the evolution of the PL spectra for untreated CsPbBrI_2_ films and COC-treated CsPbBrI_2_ films at room temperature after being exposed to low and high excitation intensities of the green light irradiation (CW 532 nm) for 30 min. In Figure 2a–2b, the time dependence of the PL spectra in the untreated CsPbBrI_2_ films was monitored at 400 and 800 μW, respectively. In Figures 2c–2d, the time dependence of the PL spectra for COC-treated CsPbBrI_2_ films was tracked at 60 and 800 μW, subsequently. Note that COC-treated films were exposed to lower excitation power than were untreated films. Specifically, in Figures 2a–2c, although the excitation intensity used for the COC-treated CsPbBrI_2_ films was four times less than that for untreated CsPbBrI_2_ films, the final count rate for the PL emission was twice as high. In Figures 2b–2d, the final count rate for PL spectra in the COC-treated CsPbBrI_2_ films was five times higher as compared with untreated CsPbBrI_2_ films even after being subjected to the same excitation intensity at 800 μW. This has been linked to asymmetric kinetic ion migration dependent nonlinearly on the excitation intensity.^17^ Thus, it is possible that excitation intensity threshold induced halide segregation is reduced, which in turn leads to the high emission intensity of the COC-treated CsPbBrI_2_ films. Such a reduction was also attributed to the average grain size, which tends to increase the energy associated with exchanging Br with I within phase-segregated domains, an increase in the carrier lifetime or an expansion of the geometrical volume linked to carrier diffusion lengths in the COC-treated films, which will be discussed in the time-resolved photoluminescence (TRPL) section.^16,17,32,33^

First, the initial emission peak of untreated CsPbBrI_2_ films was centered at 630 nm at 400 μW and 800 μW, in Figures 2a–2b and S3a–S3b. However, the initial emission peak of COC-treated CsPbBrI_2_ films at 60 W exhibited redshifts at 645 nm, in Figure 3c and S3c, attributed to the energy changes due to the interface effect between the film and COC. This also possibly occurred due to the ligands removed from the CsPbBrI_2_ film after COC treatment, which might increase the dielectric constant of the surrounding medium, consequently increasing the absorbance and giving slight redshifts.^34−36^ In addition, the initial emission peak of COC-treated CsPbBrI_2_ films at 800 μW exhibited a higher redshift of 670 nm, in Figure 3d and S3d, suggesting a strong coupling involving energy transfer by the interface effect between the CsPbBrI_2_ and the COC polymer.^36^ Second, the final emission peak of untreated CsPbBrI_2_ films at both excitation intensities exhibited a redshift of 55 at 685 nm with peaks at 630 nm emerging in Figures 2a–2b and S3a–S3b, indicating I-rich and Br-rich domains, respectively.^15,37−40^ Although the COC-treated CsPbBrI_2_ films showed similar behaviors, the final PL time series exhibited fewer redshifts of 40 nm at 60 μW and 15 nm at 800 μW at 658 nm in Figures 2c–2d and S3c–S3d, respectively. Also, I-rich domain nucleation was slowed down within 800 s at 60 μW and 800 μW compared to the untreated CsPbBrI_2_ films, suggesting a minimizing of the funnelled energy from ion migration along grain boundaries or a surface defect due to a blocking effect via COC.^1,41^

In Figures 2b and 2d, the increase in the excitation intensity showed a positive correlation with the halide demixing and the reduction of PL count rates, indicating a significant valence band edge difference. Prominent halide demixing was observed in CsPbBrI_2_ films at 800 μW. In contrast, the COC-treated CsPbBrI_2_ exhibited less of a halide demixing effect, signifying higher entropic stabilization. To get a deeper insight, temperature-dependent PL spectra were measured for both films to calculate the activation energy using the Arrhenius model illustrated in Figures 5a–5b and 6a–6b. The activation energy for the COC-treated CsPbBrI_2_ was found to be higher at 28 meV, while that of the CsPbBrI_2_ film was estimated to be lower at 20 meV. In this regard, the lower activation energy of charge carriers can overcome the larger activation barrier of thermal mixed halides under high excitation intensity. The activation energy for the halide ion migration in COC-treated CsPbBrI_2_ films is higher than that in COC-treated CsPbBrI_2_ films, reflecting the fact that halide ions exhibit fewer contributions to ionic conductivities according to the reverse order of the activation energies. This also suggests that the number of vacancies for halide ions in the COC-CsPbBrI_2_ films is relatively lower than in the CsPbBrI_2_ films because of the low formation energy, inevitably showing the fastest ion migration upon continuous illumination. This can in turn increase the concentration of charge carriers and halide vacancies accumulating and funnelling into the I-rich domain, leading to an increase in lattice distortion and more polaron formation.^14,16,18−20,40^ Note that the COC-treated film segregated faster within 1 s than the untreated film. It is hypothesized that more photoexcited carriers are generated at higher excitation intensity, strongly coupled to the perovskite lattice, forming a larger polaron size. Thus, the accumulation of strain energy might be higher or exceed the strain tolerance threshold of the COC-treated CsPbBrI_2_ lattice.^37,38,41^

It has been suggested that when the grain size is equal to or larger than 46 ± 7 nm, the grain size can exceed the diffusion length. However, if the grain size is smaller than the threshold value, the electron and hole are confined and recombine radiatively.^30,31^ To support the comparability between the grain size and charge diffusion length (*L*), TRPL measurements were used for both films at room and cryogenic temperatures. Figures 4a–4b represent the TRPL spectra fitted by two and three exponential decay functions for samples at RT and 4.2 K, respectively, corrected for the instrument response function of our detection system to evaluate the excitation and recombination processes.
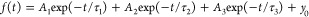
Where τ_1_ refers to short-lived PL emission contributing to band-edge exciton recombination, and τ_2_ and τ_3_ represent the long-lived lifetime attributed to trapping states arising from recombination in CsPbBrI_2_ films with a photoinduced trapped pathway.^42^ The fitted TRPL decay parameters are summarized in Table 1.

**Figure 4:**
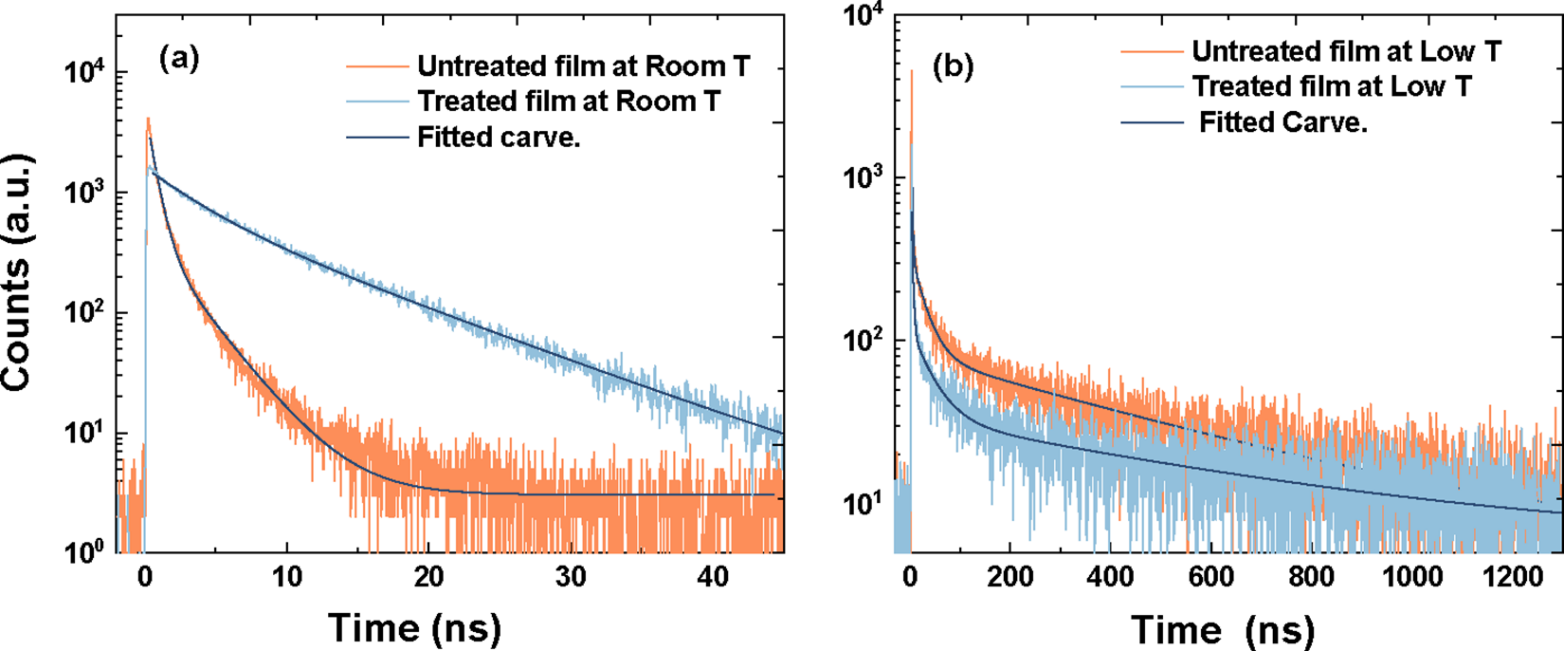
Time-resolved PL spectroscopy for CsPbBrI_2_ thin films at an excitation wavelength of 400 nm and laser power intensity of 20 μW: (a) RT untreated and COC-treated CsPbBrI_2_ films and (b) 4.2 K untreated and COC-treated CsPbBrI_2_films.

**Table 1:** Time Decay of a CsPbBrI_2_ Thin Film and COC-Treated CsPbBrI_2_ Films Fitted by Three Exponential Decay Functions Corrected for the Instrument Response Function of Our Detection System to Evaluate the Excitation and Recombination Processes

Film	τ_1_ (ns)	*A*_1_	τ_2_ (ns)	*A*_2_	τ_3_ (ns)	*A*_3_
RT CsPbBrI_2_	0.49 ± 0.02	3.48 ± 0.06	2.84 ± 0.08	0.57 ± 0.04	–	–
RT CsPbBrI_2_ with COC	4.28 ± 0.33	0.69 ± 0.05	10.67 ± 0.28	0.57 ± 0.05	–	–
4.2 K CsPbBrI_2_	23.3 ± 8.0	2.72 ± 0.39	5420 ± 750	0.055 ± 0.007	384 ± 160	0.38 ± 0.12
4.2 K CsPbBrI_2_ with COC	74 ± 5.7	3.86 ± 0.25	8000 ± 900	0.097 ± 0.011	734 ± 77	0.372 ± 0.06

The PL lifetime was found to be short in the untreated CsPbBrI_2_ with τ_1_ = 0.49 ns and τ_2_ = 2.8 ns resulting from fast charge funnelling into I-rich phases^45^ as also confirmed in figures S5a–S5b. In contrast, COC-treated CsPbBrI_2_ exhibited a longer lifetime within τ_1_ = 4.28 ns and τ_2_ = 10.67 ns, indicating COC-limiting charge funnelling into I-rich phases and defect-filling effects.^32^ This means that the electrons/holes diffuse with their diffusion length in the untreated film (180 nm) while they are confined in grains in the COC-treated films (61 nm), which was also observed by Hu and Gualdron-Reyes.^30,31^ In light of observations based on thermodynamics^46^ and strain models,^16,40^ the initial ion distribution of CsPbBrI_2_ films seems relatively homogeneous under prolonged illumination, generating holes and electrons, which quickly recombine, leading to PL emission. However, it is conjectured that the weakly bound electron and hole might dissociate, traveling with a long lifetime, causing lattice deformation through phonon coupling (polaron), and inducing halide anion redistribution. In other words, the imbalance of the Pb/I ratios on the CsPbBrI_2_ film surface generates halide vacancies or undercoordinated Pb^2+^, Cs^2+^, and I^–^ ions, increasing the chance of redox reactions. In this regard, the COC polymer matrix strongly interacts with Pb^2+^ to cover the perovskite surface/grain boundaries, improving the stability of CsPbBrI_2_ films and, therefore, reducing the iodine vacancies and suppressing the halide ion mobility in CsPbBrI_2_ films. Thus, the passivation process involving COC not only resulted in interaction with unliganded Pb^2+^ ions, leading to a reduction in surface defect density but also exerted control over the mobility of I^–^ ions on the surface of CsPbBrI_2_. As a result, the I/Pb ratio on the surface approached the stoichiometric ratio.^47^

Our finding is in agreement with the previous report that observed that the trapped state density decreased from 8 × 10^16^ to 6.64 × 10^16^ cm^–3^ after adding a Pb(NO_3_)_2_ methyl acetate solution to CsPbBrI_2_ films.^48^ Similarly, the PL lifetime in CsPbI_*x*_Br_3–*x*_ increased from 7.5 to 12.1 ns after 6TIC-4F treatment, confirming that nonradiative recombination was significantly suppressed.^49^ Moreover, lifetime decay in BaI_2_:CsPbBrI_2_ was prolonged from 2.01 to 16 ns, due to the suppression of nonradiative recombination pathways.^50^ It is also worth noticing that the grain boundaries (GBs) are sensitive places with higher defect concentration and nonradiative recombination, which disturb charge carrier movements (carrier mobility) by creating scattering centers and barriers. However, the increase in the number of GBs due to a reduction in the grain size after passivation with COC can improve CsPbBrI_2_ film charge carrier separation, free carrier density, collection, and then, ultimately, the flow enhancement of the local current. The increased number of GBs can also dissociate exciton states (electron–hole pairs) and then generate more free-charge carriers, which in turn strongly influence the performance of perovskite-based optoelectronic devices. Yang et al. found that GBs have equivalent or slightly longer PL lifetimes than perovskite film interiors/surfaces, suggesting that GBs are not dominating nonradiative recombination centers.^51^ In perovskite films, Yun et al. identified increased current collection in the vicinity of GBs, which they ascribed to enhanced carrier transport and separation along the GBs as well as downward band bending.^52^ In a similar trend, Ciesielski et al. discovered that PL lifetime decay was delayed in regions adjacent to the GBs due to carrier reflection in the areas close to the GBs.^53^

Interestingly, however, both the excitonic state lifetimes τ_1_ and the charge trapping state lifetimes τ_2_ for both films become much longer at 4.2 K than at room temperature, possibly due to suppression of polaron formation.^54^ It is also worth noting that both the short- and long-lived lifetimes of COC-treated CsPbBrI_2_ films increased by a factor of ∼3 over untreated CsPbBrI_2_ films, where COC can decelerate ion migration by increasing the activation energy barrier, reducing nonradiative relaxation processes and passivating trapped states,^16,33,55^ taking into account that, at lower temperatures, the overall free energy is low due to the entropic stabilization. In contrast, at higher temperatures, the enthalpy of electronic states tends to change, leading to increases in the polaron formation density.^42,56,57^ The reduction of the lattice distortion at 4.2 K tends to lower the strain energy and produce fewer halide vacancies, leading to charge carrier localization into the self-trapped states and enhanced PL intensity, as shown in Figure 3a–3d. The increase in the total free energy that stems from band gap differences, a funnelling and accumulation of photoexcited holes into the I-rich phase, a gradient strain accumulation caused by ion migration and lattice distortion were sufficiently inhibited in the COC-treated CsPbBrI_2_ films at both temperatures as seen in Figure 2c–2d and Figure 3c–3d. For this reason, we expect that COC can improve the thermodynamic and kinetic stabilization by filling the surface defects in the CsPbBrI_2_ films, optimizing grain size, lowering halide vacancy density, and reducing trap states, which help to minimize the rapid recombination of charge carriers with efficient charge transport in perovskites solar cells application.^1,6^ Therefore, the increase in lifetime decay and the PL intensity improvement for the COC-treated CsPbBrI_2_ films at both temperatures indicates that COC can effectively inhibit nonradiative recombination induced by trapped states in the untreated CsPbBrI_2_ films.^41^

In conclusion, we investigated the microscopic processes occurring during halide segregation in CsPbBrI_2_ films using combined spectroscopic measurements at room and cryogenic temperatures. We have put forward a passivation strategy for mitigating the halide migration of Br/I ions in the films by overcoating with a cyclic olefin copolymer (COC). We found that COC treatment optimizes the grain size in the threshold size range between 168 to 47.33 nm, thus increasing the activation energy barrier of ion migration from 20 to 28 meV. Room temperature time-dependent PL profiles at increasing excitation intensities showed that (i) the excitation threshold intensity induced phase-segregation decreases, (ii) the band gap difference between decreases, and (iii) the dominance of the entropy demixing halide ions driven by the kinetics of halide vacancy and polaron accumulation is reduced after coating. The entropy remixing halide ions and higher photostability against phase segregation are dominant at cryogenic temperatures (4.2 K).

## Supporting Information Available

Additional experimental details, including synthesis and optical characterization, and further data (PDF)
