# Supplementary material for: Stability of Mixed Lead Halide Perovskite Films Encapsulated in Cyclic Olefin Copolymer at Room and Cryogenic Temperatures
Source: J Phys Chem Lett. 2023 Dec 8;14(50):11333–41. doi: 10.1021/acs.jpclett.3c02733 (PMC10749468; doi:10.1021/acs.jpclett.3c02733)
Supplement: Supplementary file 1 — jz3c02733_si_001.pdf [file jz3c02733_si_001.pdf]

Stability of Mixed Lead Halide Perovskite Films Encapsulated in Cyclic Olefin Copolymer at Room and Cryogenic Temperatures

Mutibah Alanazi[1], Ashley Marshall[1,2], Shaoni Kar[1], Yincheng Liu[1,3], Jinwoo Kim[1], Henry J. Snaith[1], Robert A. Taylor*[1], and Tristan Farrow*[1]

[1]Clarendon Laboratory, Department of Physics, University of Oxford, Parks Road, Oxford, OX1 3PU, United Kingdom
[2]Helio Display Materials Ltd., Wood Centre for Innovation, Oxford, OX3 8SB
[3]Institute of Materials Research and Engineering, Agency for Science, Technology and Research (A*STAR), 2 Fusionopolis Way, Singapore 138634, Singapore
[*]Corresponding authors: tristan.farrow@physics.ox.ac.uk

Methodology

Synthesis

Materials

Caesium Iodide (CsI, 99.9% trace metals basis, Sigma Aldrich); lead bromide ($PbBr_2$, 99.998% metals basis, Alfa Aesar); lead iodide (PbI_2, 99.99%, TCI); dimethyl sulfoxide (DMSO, anhydrous, Sigma Aldrich); dimethylformamide (DMF, anhydrous, Sigma Aldrich); diethyl ether (DEE, anhydrous, Sigma Aldrich); isopropyl alcohol (IPA, technical grade, Sigma Aldrich); acetone (technical grade, Sigma Aldrich). All materials were used as-received without any further purifications and all precursors were stored and used in a N_2-filled glove box.

$CsPbBrI_2$ Thin Film Deposition

Optical characterisation

A CW laser operating at 532 nm with an output power of 3.5 μW was used to excite the $CsPbBrI_2$ film in the PL experimental system. The films were mounted in a continuous-flow helium cryostat to control the temperature from 4.2 K to room temperature 297 K. A 100× 0.7 N.A objective lens was held by a piezoelectric stage to focus the laser to a spot size of 0.7 μm and collect the PL emitted from the $CsPbBrI_2$ film. The PL emission was then directed to a spectrometer with a (0.07/ 0.15) nm spectral resolution depending upon the grating. The PL signals were detected by a cooled charge-coupled device detector. A Picoharp time-correlated single-photon counting system was used to for the TRPL measurements using the same experimental setup employing various excitation power densities. All these measurements were conducted under vacuum to preserve the $CsPbBrI_2$ films from contamination, such as degradation resulting from moisture or oxidation occurring under ambient conditions.

The following procedures were carried out in a N_2-filled glove box using anhydrous solvents. A 1 M solution of $CsPbBrI_2$ was prepared by dissolving 1 mmol of CsI, 0.5 mmol of PbI_2, and 0.5 mmol of $PbBr_2$ in 1 mL of DMF:DMSO mixture (3:1 volume ratio). The mixture was vortex mixed until no visible crystals remained in the solution and then it was left to stir until use. Glass substrates cut to 25 mm × 25 mm were cleaned using IPA and acetone, followed by 10 minutes of UV-ozone exposure. The cleaning process was done in air and the substrates were transferred to the N_2-filled glove box where they were spin-coating at the end of the UV-ozone treatment. 70 μL of $CsPbI_2$ solution was deposited on the center of the glass slide, then the spin-coating process was begun. The spin-coater was programmed to spin at 1000 rpm for 10 seconds, then 5000 rpm for 50 seconds. A timer was started along with the spin program and 350 μL of DEE was pipetted onto the center of the glass substrate when the timer reached 37 seconds. The substrate was then transferred to a hotplate pre-heated to 32 °C and dried for 10 minutes or until dark brown. The films were annealed at 250 °C for 10 minutes following the drying. Samples were cut down to 5 mm × 5 mm for loading into the cryostat for measurements.

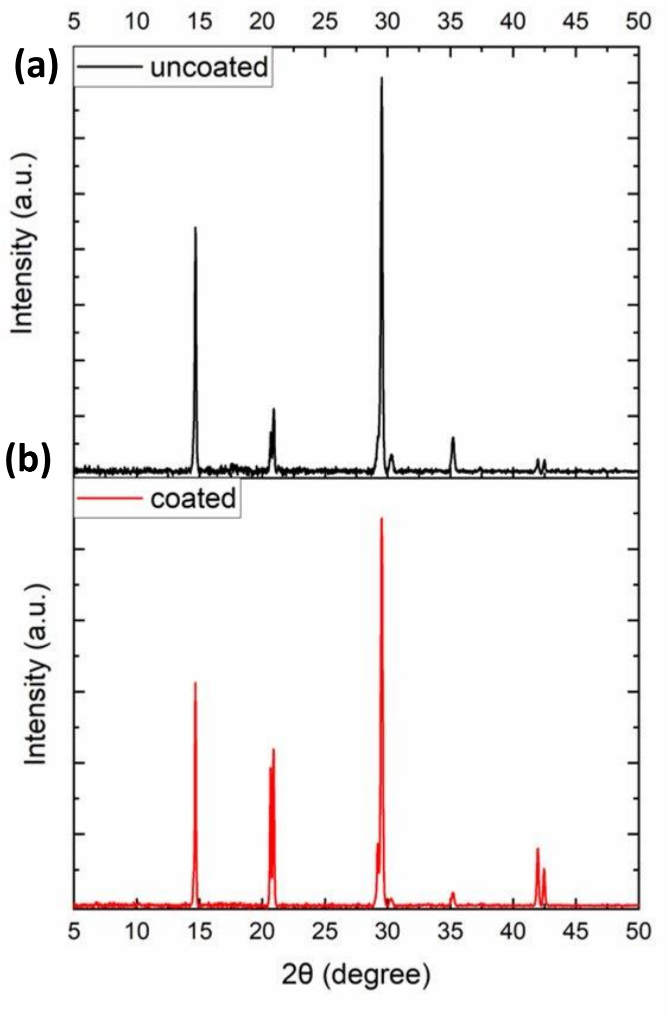

Figure S1. Room temperature X-ray diffraction (XRD) patterns of (a) the untreated $CsPbBrI_2$ films (b) the COC-treated $CsPbBrI_2$ films.

Sample name	τ_1 (ns)	A1	τ_2(ns)	A2	τ_3(ns)	A3
COC-Treated $CsPbBrI_2$ 4.2 K	74 ± 5.7	3.86 ± 0.25	8000 ± 900	0.10 ± 0.01	734 ± 77	0.73 ± 0.60
COC-Treated $CsPbBrI_2$ 100 K	7.3 ± 2.3	33 ± 5.7	50 .6 ± 9.5	5.7 ± 1.4	395 ± 30	0.35 ± 0.05
COC-Treated $CsPbBrI_2$ 150 K	26 ± 1.3	4.77 ± 0.11	110.1 ± 1.8	1.20 ± 0.05	-	-
COC-Treated $CsPbBrI_2$ 200 K	10.32 ± 0.44	2.61 ± 0.07	29.90 ± 0.25	2.97 ± 0.07	-	-
COC-Treated $CsPbBrI_2$ 250 K	5.50 ± 0.13	3. 03 ± 0.07	16.50 ± 0.20	2.66 ± 0.07	-	-
COC-Treated $CsPbBrI_2$ 290 K	4.28 ± 0.33	0.68 ± 0.05	10.68 ± 0.28	0.58 ± 0.05	-	-

Table S1. Fitting parameters of the decay times of COC-Treated $CsPbBrI_2$ films as a function of the temperature using a pulsed laser operating at 405 nm and fixed excitation intensity of 2 μW.

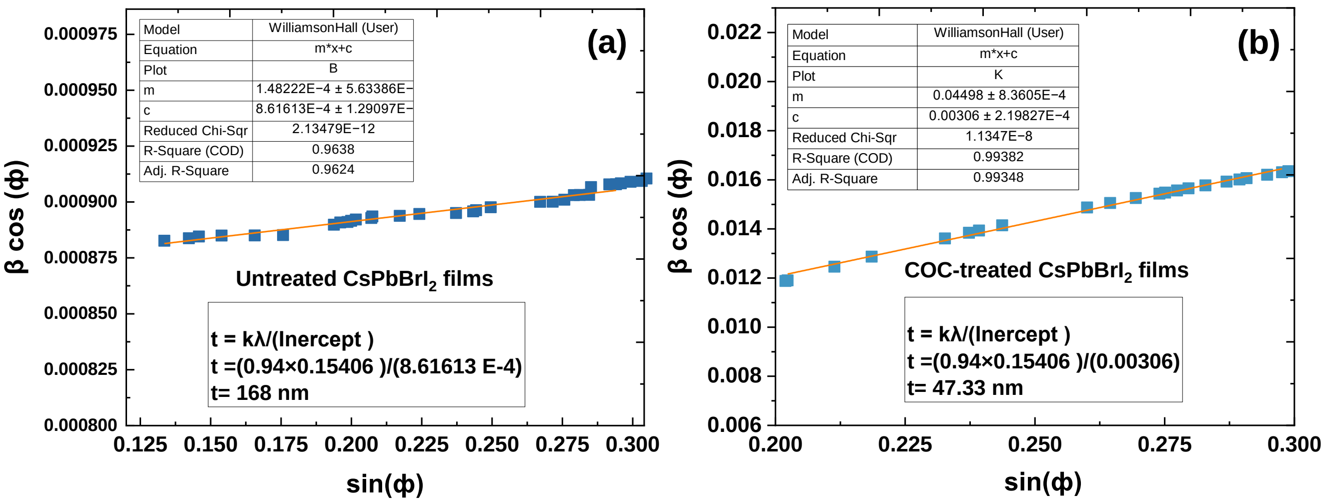

Figure S2. Williamson-Hall Plot of the XRD data using the Rietveld refinement method where the slope is a macrostrain and the intercept is a crystallite size after using the Debye-Scherrer Equation (a) untrated $CsPbBrI_2$ films (b) COC-treated $CsPbBrI_2$ films

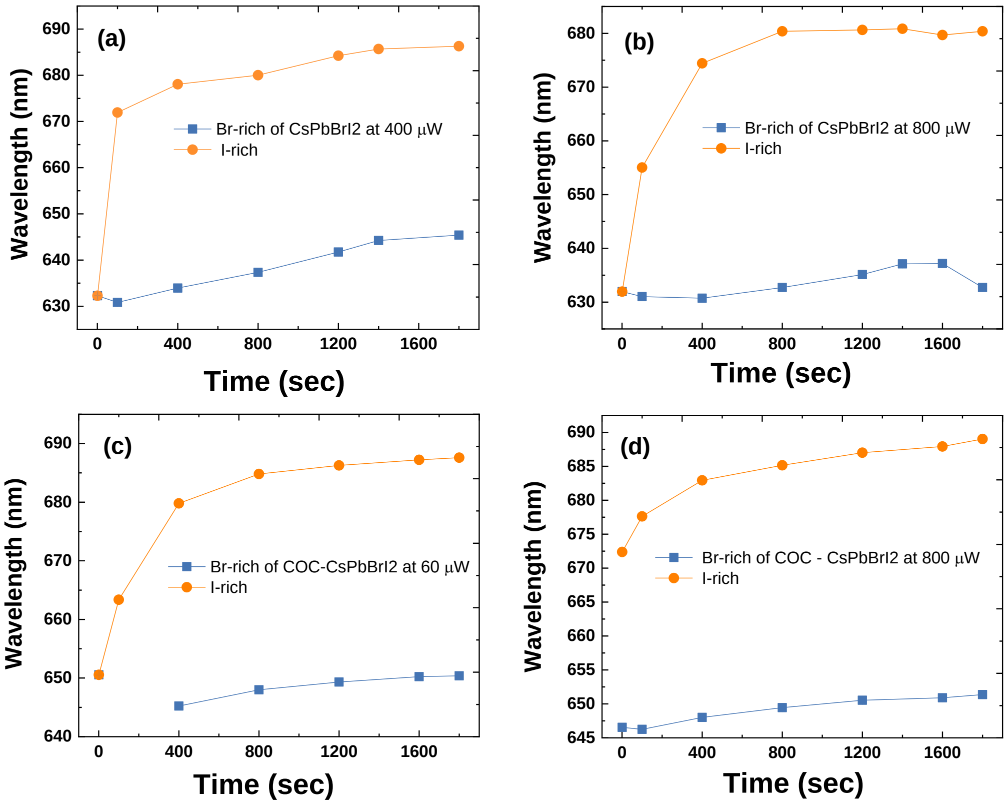

Figure S3. The rate of wavelength changes (the band gap difference from the initial mixed to final segregation states) for both untreated $CsPbBrI_2$ films and COC-treated $CsPbBrI_2$ films at room temperature after being exposed to low and high excitation intensities of the green light irradiation (CW - 532 nm) in 18000 seconds (a)(b) untreated $CsPbBrI_2$ films and (c)(d) in COC-treated $CsPbBrI_2$ films.

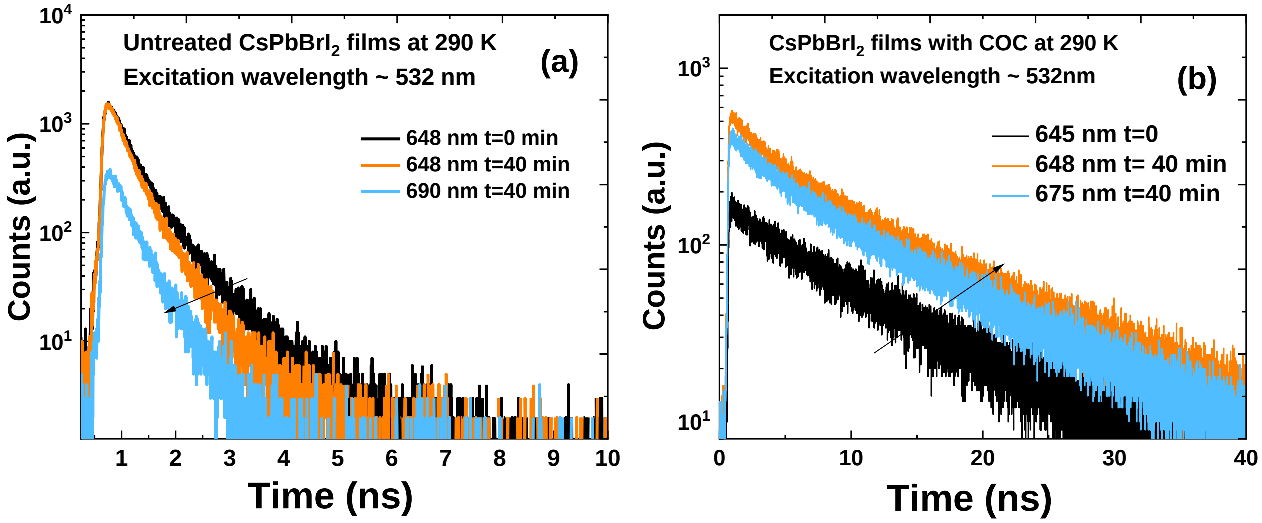

Figure S4. Room temperature time-resolved PL spectroscopy before and after phase segregation for a $CsPbBrI_2$ thin film at an excitation wavelength of 532 nm (a) TRPL of the untreated $CsPbBrI_2$ films with laser power intensity 90 μW and 11 MHz leaser repetition rate (b) TRPL of the COC-treated $CsPbBrI_2$ films with laser power intensity 10 μW and 11 MHz leaser repetition rate.

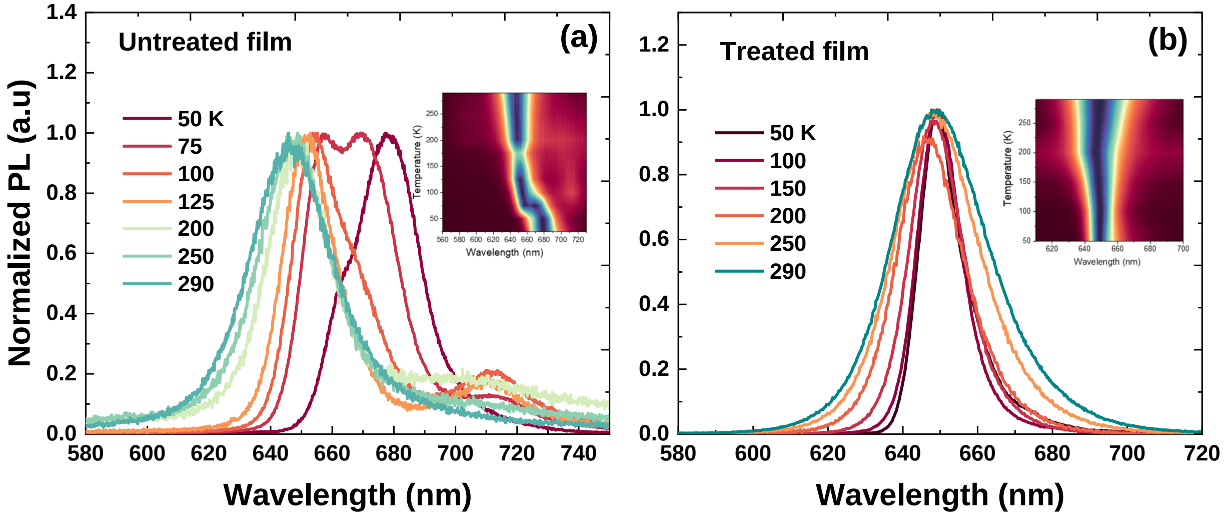

Figure S5. Temperature-dependent PL intensities of the $CsPbBrI_2$ before and after film treatment by COC in the temperature region (4.2 K to 290 K). (a) An anomalous decrease in the integrated PL intensity of untreated $CsPbBrI_2$ is generally observed at 200 μW when a 400 nm excitation is applied. (b) A photo stable temperature-dependent PL intensity is observed at 200 μW under excitation with a 400 nm laser.

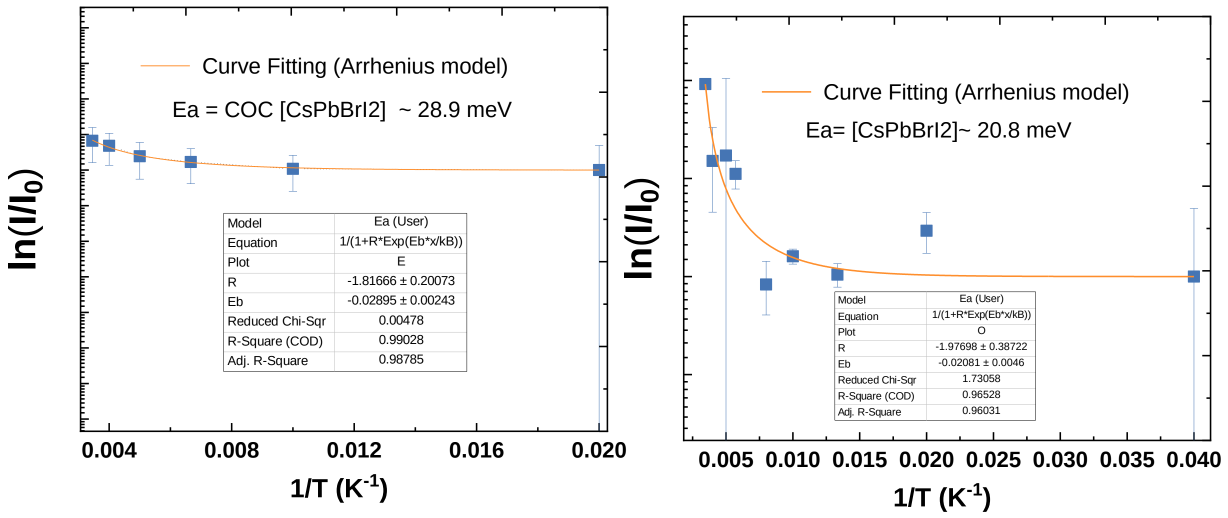

Figure S6. The PL intensity's natural logarithm as a temperature function for both untreated $CsPbBrI_2$ films and COC-treated $CsPbBrI_2$ films. The blue dots denote experimental data, and the solid lines fit the data using the Arrhenius model. the activation energy of untreated $CsPbBrI_2$ films is 20 meV and (b) the acctivation energy of the COC-treated $CsPbBrI_2$ films is 28 meV.

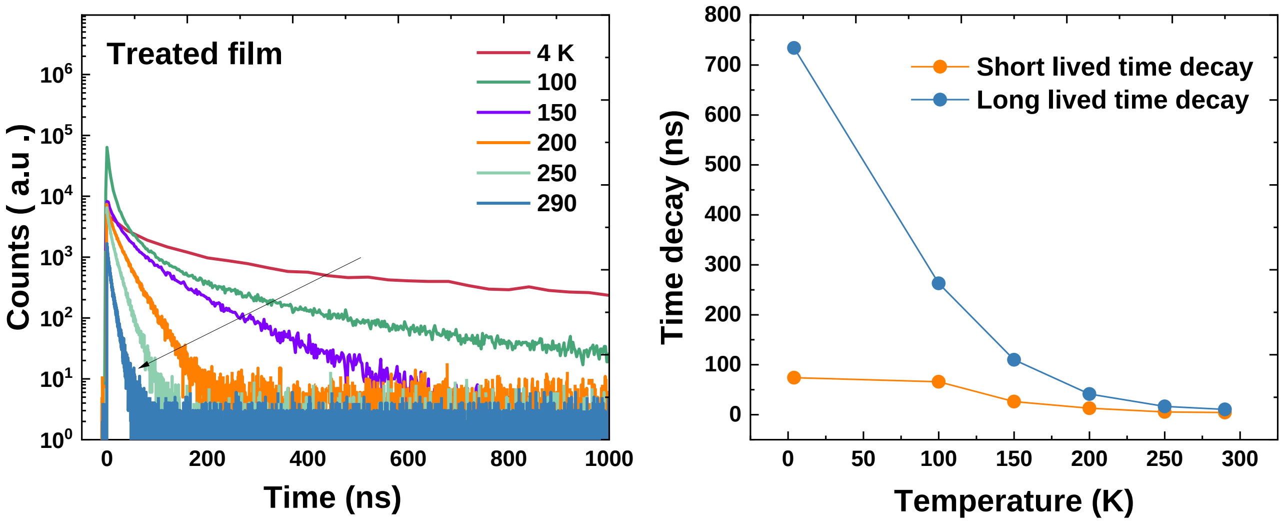

Figure S7. Temperature dependent time resolved photoluminescence spectra of the COC-treated $CsPbBrI_2$ films at 200 μW under excitation with a 400 nm laser.
